# Development of the ‘REadiness SElf-assessment (RESEA) guide’ to assist low and middle-income countries with establishing safe and sustainable radiotherapy services: a pragmatic sequential mixed qualitative methods project

**DOI:** 10.1186/s12913-021-06274-x

**Published:** 2021-03-23

**Authors:** Andrew Donkor, Tim Luckett, Sanchia Aranda, Verna Vanderpuye, Jane L. Phillips

**Affiliations:** 1 IMPACCT (Improving Palliative, Aged and Chronic Care through Clinical Research and Translation), Faculty of Health, University of Technology Sydney, Sydney, NSW Australia; 2 National Centre for Radiotherapy, Korle-Bu Teaching Hospital, Accra, Ghana; 3 Faculty of Health and CEO, Cancer Council Australia, University of Technology Sydney, Sydney, NSW Australia

**Keywords:** Radiotherapy, Readiness, Sustainable, Low and middle-income countries, Assessment, Establishment

## Abstract

**Background:**

Improving access to radiotherapy services in low and middle-income countries (LMICs) is challenging. Many LMICs’ radiotherapy initiatives fail because of multi-faceted barriers leading to significant wastage of scarce resources. Supporting LMICs to self-assess their readiness for establishing radiotherapy services will help to improve cancer outcomes by ensuring safe, effective and sustainable evidenced-based cancer care. The aim of the study was to develop practical guidance for LMICs on self-assessing their readiness to establish safe and sustainable radiotherapy services.

**Methods:**

The Access to Radiotherapy for Cancer treatment (ARC) Project was a pragmatic sequential mixed qualitative methods design underpinned by the World Health Organisation’s ‘Innovative Care for Chronic Conditions Framework’ and ‘Health System Building Blocks Framework for Action’ conceptual frameworks. This paper reports on the process of overall data integration and meta-inference from previously published components comprising a systematic review and two-part qualitative study (semi-structured interviews and a participant validation process). The meta-inferences enabled a series of radiotherapy readiness self-assessment requirements to be generated, formalised as a **RE**adiness **SE**lf-**A**ssessment (RESEA) Guide’ for use by LMICs.

**Findings:**

The meta-inferences identified a large number of factors that acted as facilitators and/or barriers, depending on the situation, which include: awareness and advocacy; political leadership; epidemiological data; financial resources; basic physical infrastructure; radiation safety legislative and regulatory framework; project management; and radiotherapy workforce training and education. ‘Commitment’, ‘cooperation’, ‘capacity’ and ‘catalyst’ were identified as the key domains enabling development of radiotherapy services. Across these four domains, the RESEA Guide included 37 requirements and 120 readiness questions that LMICs need to consider and answer as part of establishing a new radiotherapy service.

**Conclusions:**

The RESEA Guide provides a new resource for LMICs to self-assess their capacity to establish safe and sustainable radiotherapy services. Future evaluation of the acceptability and feasibility of the RESEA Guide is needed to inform its validity. Further work, including field study, is needed to inform further refinements. Exploratory and confirmatory factor analyses are required to reduce the data set and test the fit of the four-factor structure (commitment, cooperation, capacity and catalyst) found in the current study.

**Supplementary Information:**

The online version contains supplementary material available at 10.1186/s12913-021-06274-x.

## Background

The global burden of cancer is increasing, and this is even more apparent in low and middle-income countries (LMICs). Implementing evidence-based cancer care, particularly essential radiotherapy services, in LMICs is challenging. It is estimated that at least half of cancer patients will require radiotherapy to cure, improve local tumour control, achieve symptom control or improve their quality of life [1–3]. Access to radiotherapy is a basic human right for people affected by cancer.

Improving radiotherapy services is a crucial part of achieving the Sustainable Development Goals in LMICs, but unfortunately, it has largely been neglected. Like other cancer care interventions, access to radiotherapy services varies based on human development index and gross domestic product [4]. Only a third of low-income countries, half of lower middle-income countries and two-thirds of upper middle-income countries have functioning radiotherapy services [5]. The problem is compounded by: a radiotherapy workforce shortage; high initial cost that includes equipment and training, which could be amortised in a relatively short period of years; poor quality of radiotherapy services; and inefficient maintenance of radiotherapy equipment [2, 6].

A systematic review by the current authors identified that establishing safe and sustainable radiotherapy services in LMICs is a complex and technically difficult undertaking dependent on a positive policy environment, healthcare organisation and community factors [7]. Policy-level barriers include: lack of legislative and regulatory frameworks; lack of cancer control policies and plans; out-of-date epidemiological data; competing priorities for government resources; and lack of training for the radiotherapy workforce. Healthcare organisation and community-level barriers have been documented as: lack of expertise and failure to engage with relevant stakeholders including community input [7].

Assessing readiness prior to the establishment of a new radiotherapy service can improve safety, quality and sustainability [8]. There is a drive internationally for LMICs to be empowered to steer their own development programs whenever possible, rather than have these led by organisations from high income countries [1, 9]. The ‘**A**ccess to **R**adiotherapy for **C**ancer treatment (ARC) Project sought to better understand the barriers and facilitators to establishing radiotherapy services in LMICs and provide practical guidance for LMICs to self-assess their readiness to establish safe and sustainable radiotherapy services. The ARC Project began with the assumption that establishing and sustaining a new radiotherapy service consists of multiple complex processes that overlap. Also, it was assumed that radiotherapy services are the core around which other cancer services are built. Hence, a successfully established radiotherapy service can be an important mechanism for other aspects of cancer care implementation [5, 10].

### Aim

To develop practical guidance for LMICs on self-assessing their readiness to establish safe and sustainable radiotherapy services.

## Methods

### Design and conceptual framework

A meta-inference was conducted of previously published qualitative data generated by the ARC Project [7, 11, 12]. A meta-inference yields important information for generating comprehensive conclusions to inform the development and implementation of an action plan [13]. The ARC Project adopted a pragmatic sequential mixed qualitative methods design [14–16], which involved: a systematic review [7]; a series of semi-structured interviews [11]; and a participant validation process [12]. A mixed methods design was adopted because it enabled a deeper understanding of the broader set of challenges to establishing new radiotherapy services [17].

Two conceptual frameworks provided by the World Health Organisation’s (WHO) guided the ARC Project, namely the: Innovative Care for Chronic Conditions Framework; and Health System Building Blocks Framework for Action [18, 19]. Both frameworks have been applied in several countries to strengthen chronic care delivery [20–22]. Ethical approval was obtained from the Human Research Ethics Committee at the University of Technology Sydney, Australia, and all participants gave verbal informed consent. The ARC Project was conducted between October 2016 and May 2020.

Detailed methods and results for each of the studies are described elsewhere [7, 11, 12], but are briefly summarised as follows.

### Systematic review

**Fig. 1:**
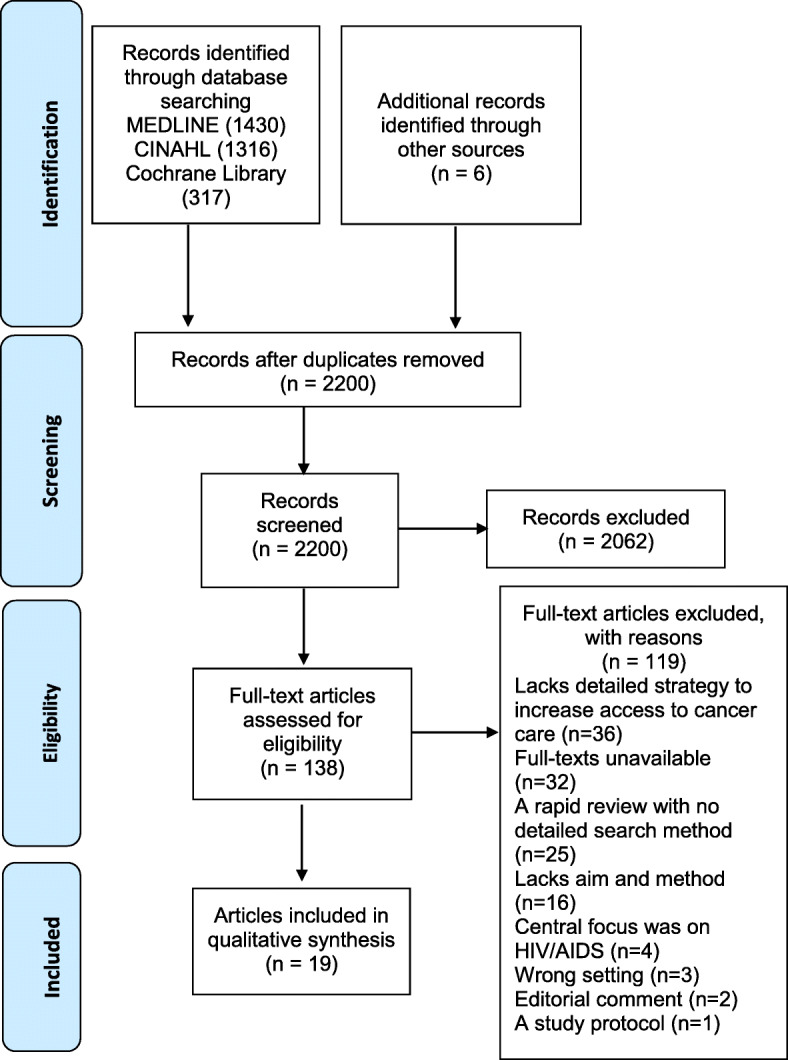
PRISMA Flow diagram illustrating study search and selection

The systematic review appraised strategies adopted by LMICs to improve access to cancer treatment and palliative care services, as well as identified the facilitators and barriers that contributed to the successful implementation of cancer improvement strategies in LMICs [7]. Three electronic databases (CINAHL, MEDLINE and Cochrane Library) were searched using terms related to: cancer; cancer treatment; improvement strategies; and LMICs. Reference lists of articles were examined to identify potentially relevant studies (Fig. 1). Data were extracted from included studies into a standardised data extraction form. A narrative synthesis using approaches described by Popay and colleagues [23] was adopted. Included studies were independently coded by two reviewers (AD and TL) and strategies mapped against the WHO’s Innovative Care for Chronic Conditions Framework.

### Semi-structured interviews

Semi-structured interviews explored global radiotherapy experts’ experiences and identified perceived barriers and facilitators to establishing safe and sustainable radiotherapy services in LMICs [11].

Interview participants were global radiotherapy experts, including 11 radiation oncologists, three medical physicists, two radiation therapists and an administrator, with experience in establishing and/or sustaining radiotherapy services in LMICs, such as Kenya, Ethiopia, Egypt, Brazil, Jordan, India, Nepal, Peru and Zambia. All the participants were able to communicate in English.

A non-probability approach was used in sampling participants. Participants were recruited from the authors of relevant studies included in the earlier systematic review [7], international radiotherapy reports [24, 25], recommendations, personal and professional networks. Recruitment was undertaken by AD, with an email introduction by senior team members (JP or SA).

### Participant validation process

The participant validation process obtained feedback from seven global radiotherapy experts about the utility of the potential radiotherapy service development **RE**adiness **SE**lf-**A**ssessment (RESEA) Guide for use by LMICs [12]. A draft RESEA was distributed to the global radiotherapy experts who participated in the semi-structured interviews via email to confirm or refute its relevance and to assist with its refinement. Participants were asked if they agree with the generated radiotherapy development requirements within the RESEA Guide by addressing the following statements: i) if you think any of the requirements already listed should be removed, please place “R” as “Remove”; ii) if you think any of the requirements already listed should be included but needs to be revised or modified in some way, please place “M” as “Modify”; and iii) if you think any of the requirements already listed should be included without changes, please place “A” as “Accept”.

**Fig. 2:**
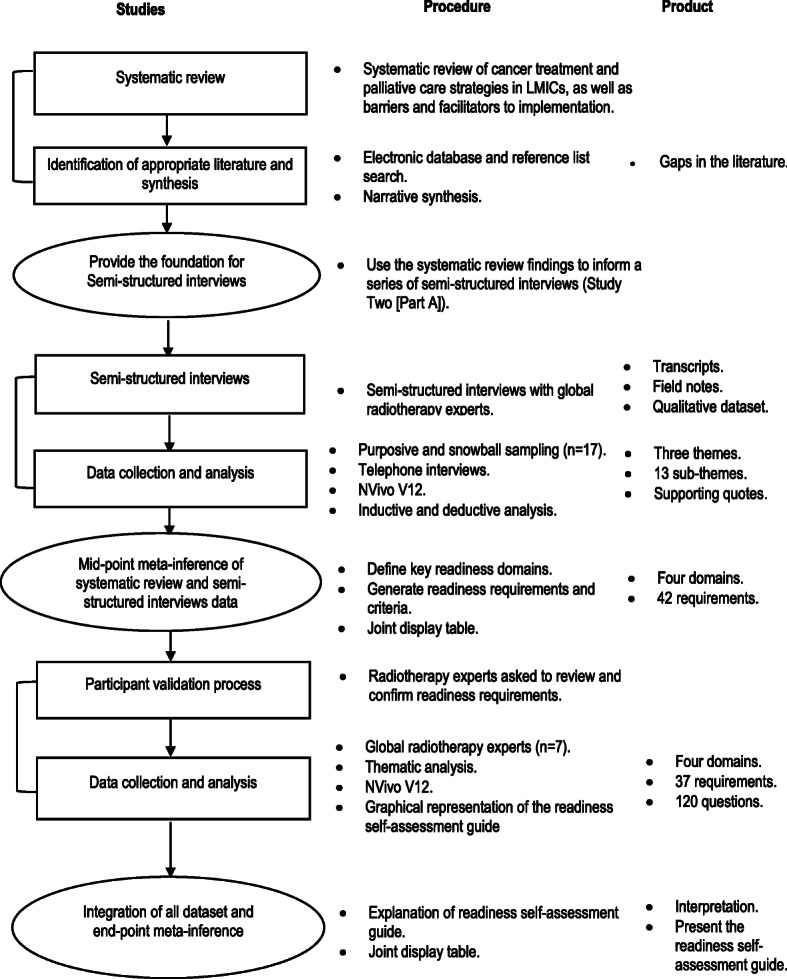
Sequential mixed qualitative methods design for the ARC Project. Adapted from [17] and [26]

Figure 2 outlines the various studies, procedures and related products of the ARC Project.

### Data analysis

The ARC Project answered three different questions about cancer care improvement strategies, implementation barriers and facilitators and radiotherapy readiness requirements. Table 1 shows the research questions answered by the findings of relevant studies. In this paper, the mid-point meta-inference answers research question two while the end-point meta-inference answers the research question three by integrating all the ARC Project’s data. Mid-point meta-inference was defined as the conclusion generated through an integration of the interpretations that were obtained from the findings of the systematic review and the series of semi-structured interviews [13]. Likewise, end-point meta-inference refers to the meaning-making process and product created by synthesising all the ARC Project’s data [i.e. systematic review, semi-structured interviews and participant validation process [7, 11, 12]].

**Table 1:** The ARC Project’s research questions, studies and objectives

Research questions	Studies and objectives
1. What efforts have been made to improve cancer care in LMICs and how effective have they been?	•Systematic review appraising strategies adopted by LMICs to improve access to cancer treatment and palliative care services [7].
2. What are the barriers and facilitators to establishing safe and sustainable radiotherapy services in LMICs?	•Systematic review appraising facilitators and barriers that contribute to the successful implementation of cancer treatment and palliative care improvement strategies in LMICs [7].
	•Semi-structured interviews identifying and describing barriers and facilitators to establishing and sustaining radiotherapy services in LMICs [11].
	•Mid-point meta-inference integrating data from the systematic review and semi-structured interviews to gain a deeper understanding of the barriers and facilitators that need to be considered by LMICs planning to develop a local radiotherapy service
3. What readiness requirements do LMICs’ need to consider when setting out to establish safe and sustainable radiotherapy services?	•Participant validation process seeking feedback from global radiotherapy experts about the utility of the potential radiotherapy service development readiness assessment requirements identified via the mid-point meta-inference [12].
	•Data integration and end-point meta-inference; and presentation of the ‘REadiness SElf-Assessment (RESEA) Guide’ for LMICs establishing safe and sustainable radiotherapy services.

The integration of the ARC Project’s data either mid-point or end-point occurred through three approaches, namely: integration through narrative; integration through data transformation; and integration through a joint display [27]. For the mid-point meta-inference, theme-by-theme approach was used to narratively integrate findings from a systematic review [7] and perspective of global radiotherapy experts [11] to gain deeper understanding into the barriers and facilitators to establishing safe and sustainable radiotherapy services in LMICs.

For the end-point meta-inference, all the ARC Project’s data [7, 11, 12] were transformed into readiness domains, requirements and questions through an iterative process of team discussion, revision, team input and content analysis. Also, all the ARC Project’s data were integrated by organising the data into joint display table and visual display to confirm, as well as draw out new or enhanced insights beyond the information gained from the separate studies within the ARC Project.

### Findings

Emerging from the ARC Project’s meta-inferences is a deeper understanding of the: i) barriers and facilitators to establishing safe and sustainable radiotherapy services in accordance with the WHO’s Innovative Care for Chronic Conditions Framework and Health System Building Blocks Framework for Action; and ii) the radiotherapy readiness requirements for LMICs as described in the RESEA Guide. Each of these outputs are summarised below:

i. **Barriers and facilitators to establishing safe and sustainable radiotherapy services**

A large number of factors acting as facilitators were also reported to be barriers, depending on the situation. This reality was most evident in relation to: awareness and advocacy; political leadership; epidemiological data and integrated cancer control policy; financial resources; basic physical infrastructure; radiation safety legislative and regulatory framework; project management; and radiotherapy workforce training and education.

#### Awareness and advocacy

The integrated data provided new insights into barriers to creating awareness and advocacy, which included: lack of coordination among advocates; resource constraints; local champions’ lack of power to convince political leaders; and negative attitude towards cancer and its treatment, particularly radiotherapy services. It emerged that managing these barriers required an ability to harmonise civil society organisations and/or individuals with similar priorities to prevent and reduce duplication of effort.

#### Political leadership

It was recognised that a central factor for mobilising and allocating resources was gaining commitment from political leadership. A deeper understanding was gained into: political conflicts; a lack of continuity of leadership; endemic bureaucratic corruption; competing demands for scarce resources; and misinformation about the feasibility of radiotherapy services in LMICs, which present a significant negative impact on health system. Two main political leadership facilitators for gaining commitment to establish a new radiotherapy service were identified: ministerial endorsement or approval of a radiotherapy service implementation plan; and high-profile figure having been diagnosed with or dying from cancer. Ministers responsible for health are key policy and decision-makers who are able to influence healthcare budgeting; therefore, their support often reduces political opposition to establishing a radiotherapy service.

#### Epidemiological data and integrated cancer control policy

It was evident that defining any LMIC’s cancer profile was reliant on accurate epidemiological data extracted from routine information collected at the local, regional, national and/or international levels. Yet many LMICs lack population and/or hospital-based cancer registries so have access to very limited cancer epidemiological data, which can lead to them making poor decisions. Conversely, the critical role of civil society organisations, international organisations and/or agencies in improving the availability and quality of cancer registries in LMICs was acknowledged.

#### Financial resources

The integrated data confirmed several financial barriers, including: prohibitive cost of the radiotherapy infrastructure; lack of comprehensive and reliable line-item budget; lack of a public-private partnership legal framework; discontinuity of political leadership; and failure to justify the financial viability of a radiotherapy service. It is important to develop a compelling and credible business case, not just a moral case, to gain funds from governmental budget.

#### Basic physical infrastructure

Lack of reliable supply of electricity and water and poor road network were identified as basic physical infrastructure barriers to establishing safe and sustainable radiotherapy services in most LMICs. However, implementing cancer patient assistance programmes such as accommodation, transportation, financial assistance and solar-powered radiotherapy were recognised as facilitators that enable more cancer patients to access radiotherapy services and at a reasonable cost.

#### Radiation safety legislative and regulatory framework

The integrated data offers new insight into the importance of developing a legislative and regulatory framework to ensure the radiotherapy service meets international and national radiation safety and protection standards. All LMICs are entitled to leadership and technical support if they are International Atomic Energy Agency (IAEA) members. Unfortunately, some LMICs are not members of IAEA and have not been able to develop and enforce a legislative and regulatory framework for the reason that the legal process and administrative tasks are complex, requiring expertise and resources.

#### Project management

Numerous project management barriers were identified, including: lack of experience and well-informed strategic planning team members; inadequate stakeholder engagement; weak contract negotiations with powerful vendors; and inability to clearly define the roles and responsibilities of vendors, which contributes to their lack of accountability, especially in relation to cost and availability of parts, maintenance plans and in-house workforce training. Creating a multidisciplinary implementation team, appointing a responsible project manager, engaging with the IAEA technical cooperation programme and making appropriate arrangements for commissioning and licensing of the radiotherapy equipment are important facilitators.

#### Radiotherapy workforce training and education

The integrated data showed that most LMICs often overlooked the importance of building radiotherapy workforce capabilities and are not specific about timeline and budget for educational plan. In some cases, radiotherapy equipment lay idle, for lack of a prepared workforce or dependant on overseas experts to be able to operate the radiotherapy equipment in the short-term. However, it was recognised that collaborative training and educational programmes often provide peer-to-peer support, information sharing and hands-on in-country fellowships.

ii. **Radiotherapy readiness requirements as described in the RESEA Guide**

**Fig. 3:**
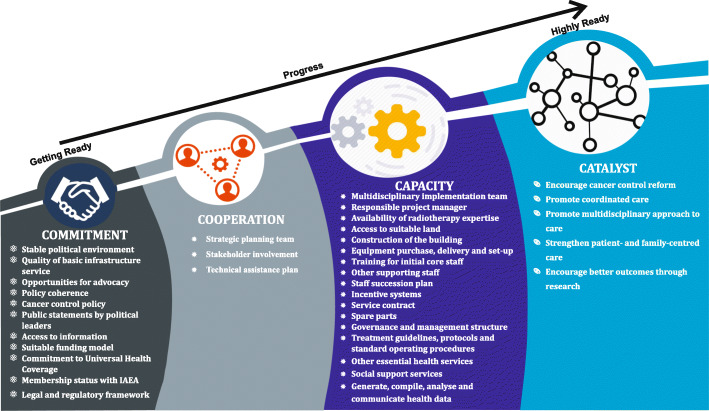
Conceptual overview of requirements across the four domains aimed at assessing LMICs’ readiness to establish safe and sustainable radiotherapy services

The radiotherapy service development ‘RESEA Guide’ for use by LMICs is the output of the ARC Project’s data integration and end-point meta-inference (see supplementary material 1). The RESEA Guide is framed around four key domains: commitment; cooperation; capacity; and catalyst. Each of these four domains summarised describes 37 requirements that ought to be completed when establishing a new radiotherapy service and includes 120 questions that need to be answered. Figure 3 presents a schematic overview of the RESEA Guide.

#### Commitment

In the RESEA Guide, commitment describes the willingness of LMICs to put in place the necessary political, policy, funding and regulatory conditions to implement a new radiotherapy service. Twelve requirements are considered important to identifying and confirming local commitment and support to establish a safe and sustainable radiotherapy service, including: the presence of a safe, stable and supportive political environment; quality of basic infrastructure service; opportunities for advocacy; policy coherence; cancer control policy; public statements by political leaders; access to information; suitable funding model; commitment to universal health coverage; membership status with the International Atomic Energy Agency (IAEA); legal and regulatory framework; and independent regulatory authority. Creating a clear vision, availability of local champions, external pressure from international agencies and desire to address radiotherapy service demands of the population all serve as leveraging mechanisms for achieving and maintaining a level of commitment within LMICs for establishing a new radiotherapy service.

#### Cooperation

Cooperation in the RESEA Guide is described as the effective involvement of relevant international, national and local stakeholders in the planning, commissioning and operationalisation of a new radiotherapy service. Three requirements were confirmed critical to identifying stakeholders’ willingness to work together to establish a safe and sustainable radiotherapy service: strategic planning team; stakeholder involvement; and technical assistance plan. It was recognised that the LMIC preparing to establish a new radiotherapy service may need to perform a stakeholder analysis to define, engage and gain better understanding of relevant stakeholders’ expectations. An important reason for developing a technical assistant plan is to avoid duplication of supports from international agencies and organisation.

#### Capacity

In the RESA Guide, capacity refers to the ability to translate both commitment and cooperation to achieve sustainable results through effective and efficient management of the radiotherapy service implementation process. Seventeen requirements underpin the identification of local capacity to implement and operationalise a new radiotherapy service: multidisciplinary implementation team; responsible project manager; availability of radiotherapy expertise; access to suitable land; construction of the building; equipment purchase, delivery and set-up; service contract; training for initial core staff; other supporting staff; staff succession plan; incentive systems; governance and management structure; treatment guidelines, protocols and standard operating procedures; other essential health services; social support services; and generate, compile, analyse and communicate health data. Project management to implement the radiotherapy service is leadership intensive, which is required to create systems for coordination.

#### Catalyst

The final domain in the RESEA Guide refers to the potential to leverage the radiotherapy service and fundamentally develop an integrated cancer service. The catalyst domain contains five requirements which were related to: encouraging cancer control reform; promoting coordinated care; strengthening patient- and family-centred care; promoting a multidisciplinary approach to care; and encouraging better outcomes through research. Catalyst stresses the importance of mobilising resources to develop a comprehensive cancer service and implementing strategies for effective communication to improve transition across specialists.

## Discussion

The RESEA Guide is the first resource to provide practical support for LMICs to self-assess their readiness to establish safe and sustainable radiotherapy services. The four domains of the RESEA Guide, commitment, cooperation, capacity and catalyst all need to be considered and addressed by LMICs when establishing a new radiotherapy service. The RESEA Guide is comprehensive enough to capture the various aspects of the radiotherapy service development and implementation process ranging from country/policy (macro) and/or service (meso) level concerns.

Establishing a new radiotherapy service without an in-depth understanding of the LMIC’s preparedness can contribute to sub-optimal outcomes. While there are several readiness assessment tools, such as: ‘Ready, Set, Change’ Readiness Support Tool [28]; e-health Readiness Assessment Tool [29]; and Organizational readiness for change [30], few are relevant for appraising LMICs’ readiness to establish safe and sustainable radiotherapy services. The ‘10-Step Framework’ [31] is considered not broad enough to cover the necessary critical success factors to enable establishment of new radiotherapy services in LMICs. The findings from the ARC Project suggest that the RESEA Guide can support LMICs to assess potential barriers and map existing opportunities by devising actionable plans and measures to eliminate inefficiencies and waste when establishing a new radiotherapy service.

Any LMIC planning a new radiotherapy service can use the RESEA Guide with major stakeholders, such as ministry of health/finance representations, clinical leadership (radiation oncologist, medical physicist and radiation therapist) and radiation safety regulator to facilitate the exchange of information and ideas. A collaborative approach is particularly important due to factors such as ensuring ongoing commitment and support by relevant stakeholders for all aspects of the radiotherapy project. The use of the RESEA Guide can potentially facilitate a consensus process, with a skilled facilitator creating a safe environment where stakeholders are able to openly express their opinions, make necessary compromises and help the group arrive at decisions acceptable to all concerned. As a ‘City Cancer Challenge’ initiative on improving cancer care services in LMICs has highlighted [32], open dialogue, co-design and stakeholder engagement are key principles of effective utilisation of the Guide. Effective stakeholder engagement and community consultations create and sustain trust, cooperation and local ownership, which facilitate the implementation of the radiotherapy service [7, 11, 31].

The RESEA Guide gives LMICs an opportunity to better plan and organise radiotherapy service implementation activities. In operational terms, the completion of the RESEA Guide could take place in a one-day workshop or a series of workshops with relevant stakeholders and/or working group members. Each stakeholder group could evaluate one domain of the RESEA Guide but scans the other domains to ensure that requirements that need long-term planning such as finance, workforce, equipment purchase, quality assurance and commission are addressed concurrently.

The working group can also proceed through the four domains of the RESEA Guide, answering each question to determine readiness and prioritise action plans. To avoid getting trapped in the details when assessing readiness, the working group ought to: read each of the questions; and consider the evidence provided to them to better inform and shape their decisions for each specific requirement for establishing a safe and sustainable radiotherapy service. It is recommended to discuss each question, reflect on strengths, identify areas for improvement and develop possible solutions for each challenge. Assessing readiness is an ongoing process; not a one-off activity [33]. Hence, the RESEA Guide is designed to be used throughout the planning, implementation and maintenance of the radiotherapy service.

### Strengths and limitations

This pragmatic sequential mixed qualitative methods project has a number of key strengths. Data were gathered by consulting widely and capturing the views and experiences of radiotherapy experts working in nine LMICs, as well as seven international radiotherapy experts who have worked with several LMICs to strengthen radiotherapy services. Rigorous mixed methods data collection and analysis combined with data integration and meta-inference have provided a deeper insight into the challenges and potential solutions to establishing safe and sustainable radiotherapy services. The ARC Project’s collaborative process led to the development of a low cost and simple RESEA Guide that could help policy-makers, planners and implementers to establish successful radiotherapy services in LMICs.

The ARC Project’s main limitation concerned the difficulty in recruiting a representative sample of radiotherapy experts working in LMICs, and in retaining all those who participated in the interviews through the validation process. In particular, no nurses were interviewed, and none of the medical physics experts who participated in the semi-structured interviews contributed to the participant validation process. As a result, the findings may not represent the experiences of the broader cancer care professionals working in LMICs. Future evaluation of the acceptability and feasibility of the RESEA Guide with a broader cross-section of professionals, organisations and agencies working on radiotherapy initiatives in LMICs is needed to inform its validity. A validated RESEA Guide could help in the assessment of preparedness and may also improve implementation of radiotherapy services in LMICs.

## Conclusions

The ARC Project has created the RESEA Guide to support LMICs’ appraise their readiness to establish safe and sustainable radiotherapy services. Importantly, the RESEA Guide offers practical support for LMICs to better understand the barriers and select appropriate risk response strategies to ensure successful establishment of new radiotherapy services. Further work, including field study, is needed to inform further refinements. Exploratory and confirmatory factor analyses are required to reduce the data set and test the fit of the four-factor structure (commitment, cooperation, capacity and catalyst) found in the current study.

## Supplementary Information

**Additional file 1: Supplementary material 1.** Radiotherapy service development RESEA Guide for use by LMICs

## Data Availability

Anonymised datasets analysed in the current study are available from the corresponding author on reasonable request.

## Abbreviations

LMICs: Low and middle-income countries
ARC: Access to Radiotherapy for Cancer
WHO: World Health Organisation
RESEA: REadiness SElf-Assessment
IAEA: International Atomic Energy Agency
