# Supplementary material for: Development of the ‘REadiness SElf-assessment (RESEA) guide’ to assist low and middle-income countries with establishing safe and sustainable radiotherapy services: a pragmatic sequential mixed qualitative methods project
Source: BMC Health Serv Res. 2021 Mar 23;21:268. doi: 10.1186/s12913-021-06274-x (PMC7988910; doi:10.1186/s12913-021-06274-x)
Supplement: Supplementary file 1 — Additional file 1: Supplementary material 1. Radiotherapy service development RESEA Guide for use by LMICs [file 12913_2021_6274_MOESM1_ESM.docx]

Supplementary material 1: Radiotherapy service development RESEA Guide for use by LMICs

**COMMITMENT**

**Domain 1**

| **Requirements** | **Questions** | **What is known (evidence)?** | **Next steps (actions)** |
| --- | --- | --- | --- |
| - 1. Safe, stable and supportive political environment | - What is the LMIC’s political and security situation? - Are there any international sanctions imposed on the LMIC? - Are there government structures and processes defined by laws to support financial accountability and transparency? |  |  |
| - 1. Quality of basic infrastructure service | - What is the nature of the road and transport system that leads to the radiotherapy centre? - How consistent is water and electricity supply? - Is there evidence of infrastructure policy for the improvement of basic infrastructure services? |  |  |
| - 1. Opportunities for advocacy | - Is there a vision that supports expanding access to radiotherapy services? - How widely is the vision to establish a radiotherapy service shared? - Is there a vision champion (e.g. civil society organisations or health professional) who is supportive of action to improve access to radiotherapy services? - Is the vision champion well equipped to lead engagement and advocacy efforts? - Are civil society organisations collectively working together? - Has the civil society organisation forged a link with international organisations? |  |  |
| - 1. Policy coherence | - Is there evidence of collaboration between government ministries and agencies to develop and implement cancer control plans? |  |  |
| - 1. Cancer control policy | - Does the LMIC have a country-wide cancer control policy? - Is the cancer control policy costed? - Is there support to implement the cancer control policy? |  |  |
| - 1. Public statements by political leaders | - Is there evidence of supportive statements by a political leader(s)/ministry of health to improve access to radiotherapy services? - Are there other things competing for the resources or time of political leaders? |  |  |
| - 1. Access to information | - Is a population-based or hospital-based cancer registry data available in the LMIC? - Is there a plan to collaborate with the International Agency for Research on Cancer (IARC) Global Initiative for Cancer Registry Development to develop a cancer registry? |  |  |
| - 1. Suitable funding model | - Is the required funding to establish the new radiotherapy available? - If not, is there evidence to support a business case for a radiotherapy service? - Is there evidence of collaboration between the ministry of health and ministry of finance in the process of developing a business case for a radiotherapy service? |  |  |
| - 1. Commitment to universal health coverage | - Is there evidence of efforts to address financial hardship at the point of radiotherapy service delivery? |  |  |
| - 1. Membership status with the IAEA | - Is the LMIC a member of the IAEA? - If not, is there evidence of the LMIC planning to establish membership with the IAEA? |  |  |
| - 1. Legal and regulatory framework | - Does the LMIC have a legal and regulatory framework? - Is the regulatory framework adequately enforced? |  |  |
| - 1. Independent regulatory authority | - Is there evidence of an independent LMIC-level regulatory authority that is supportive of efforts to establish radiotherapy service as well as ensure compliance with international standards of radiation safety, security and protection? |  |  |

**COOPERATION**

**Domain 2**

| **Requirements** | **Questions** | **What is known (evidence)?** | **Next steps (actions)** |
| --- | --- | --- | --- |
| - 1. Strategic planning team | - Has the LMIC appointed individuals to constitute the strategic planning team? - Has a comprehensive needs assessment been conducted? - If not, is there evidence of where data will be sourced for a need assessment? - Has the LMIC outlined how it will use the data from the comprehensive need assessment in the planning process? - Has the LMIC identified where it will find financial and human resources necessary to implement the radiotherapy service? - Has the strategic planning team provided a draft that outlines the strategic direction, priorities, goals and milestones as well as objectives to achieving the radiotherapy service development goal? - Has the final plan been reviewed, commented on and approved by the ministry responsible for health? |  |  |
| - 1. Stakeholder involvement | - Is there evidence of a comprehensive list of all possible stakeholders? - Does the planning team have the necessary information about the stakeholders? - If not, does the planning team know where and how information about the stakeholders can be obtained? - Does the planning team know how each stakeholder can contribute to influencing the radiotherapy project success? - Does the planning team know what constitutes satisfaction for each stakeholder? - Is there evidence of a strategy to effectively involve relevant stakeholders? |  |  |
| - 1. Technical assistance plan | - Has the LMIC identified the kind of assistance and how much such assistance will be sourced from an international agency, organisations, volunteers or consultants? - Has the LMIC listed where it can find the assistance? |  |  |

**CAPACITY**

**Domain 3**

| **Requirements** | **Questions** | **What is known (evidence)?** | **Next steps (actions)** |
| --- | --- | --- | --- |
| - 1. Multidisciplinary implementation team | - Has the right implementation team that is responsible for the day-to-day management of the radiotherapy service implementation activities been assembled? - Are the members of the implementation team clear about their roles and responsibilities? - Has a system been set-up by the implementation team to communicate, monitor, evaluate and review the progress of the radiotherapy service implementation tasks? |  |  |
| - 1. Responsible project manager | - Is there evidence of a project manager overseeing the implementation of the radiotherapy list of tasks? |  |  |
|  | - Is the radiotherapy service implementation process guided by a realistic timeframe? |  |  |
|  | - Have all the relevant stakeholders involved in implementing the radiotherapy service agreed on the timeframe? |  |  |
| - 1. Availability of radiotherapy expertise | - Is the LMIC having access to adequate technical expertise locally to implement the list of tasks outlined in the radiotherapy service development action plan? - If not, has any arrangement been made to recruit external individuals with technical expertise to perform the radiotherapy service implementation tasks? |  |  |
| - 1. Access to suitable land | - Is the land earmarked for the radiotherapy service legally registered? - If so, is there documentation of the land registration? - Is the land undergoing any form of hazard assessments? - If not, is there a plan for the land to undergo environmental and engineering assessments? |  |  |
| - 1. Construction of the building | - Are there resources to support the construction of the radiotherapy building? - Are strategies implemented to ensure construction materials undergo appropriate quality assurance evaluations? - Is there a contingency plan to minimise or avoid construction delays? - Has the independent regulator been involved in the process of constructing the radiotherapy building? |  |  |
| - 1. Equipment purchase, delivery and set-up: | - Has the LMIC purchased the right radiotherapy equipment, treatment planning system, quality assurance equipment, and other simulation and treatment accessories that are appropriate for the country? - Does the LMIC has the capabilities to install and commission the equipment? - If not, has the LMIC arranged to ensure timely installation, commissioning and licensing? |  |  |
| - 1. Service contract | - Does the LMIC has enough information as well as the necessary knowledge to confidently understand and negotiate a radiotherapy service contract? - Has the contract been written with the involvement of a trained lawyer? - Is there an agreement on a planned maintenance strategy to ensure all necessary resources such as spare parts and engineers are available to reduce equipment downtime? - Is there an arrangement on support for training radiotherapy workforce and local engineers? |  |  |
| - 1. Training for initial core staff | - Who are the core staff that will be involved in the delivery of the radiotherapy service? - How many initial core staff will be needed to effectively deliver safe and quality radiotherapy services? - What arrangement has been made to train and educate the core staff in an appropriate institution? - How much money is required for the training and education of the core staff? - Are there strategies in place for ensuring a sustainable radiotherapy workforce, with up-to-date knowledge and skills? - What are the regulatory mechanisms required to maintain quality of education, training and practice? |  |  |
| - 1. Other supporting staff | - What other human resources (such as physiotherapists and dieticians) are available in the LMIC to support the delivery of quality radiotherapy services? - Do human resources have the required skills to support the delivery of radiotherapy services? - If not, is there a plan to train them? |  |  |
| - 1. Staff succession plan | - Is there evidence of a long-term plan to establish radiotherapy specific academic institution(s) to develop and allocate radiotherapy workforce? |  |  |
| - 1. Incentive systems | - Has the LMIC established a succession plan that considers the training or appointment of radiotherapy staff? |  |  |
|  | - Is there evidence of an incentive plan to attract and create an optimal mix of radiotherapy workforce? |  |  |
| - 1. Governance and management structure | - Is there evidence of a radiotherapy governance-management structure for decision-making, administrative and executive functions? - Has a multi-stakeholder approach been used in choosing members of the radiotherapy service governance body to optimise performance? - If not, are efforts underway to appropriately define the composition of the governance and management board? - Are the roles and responsibilities of the radiotherapy service management board clearly defined? - Can the radiotherapy management board work as a team through collegial relationships that promote respect and trust? - Is there a plan to keep members of the radiotherapy management board up to date with the latest developments in radiotherapy? |  |  |
| - 1. Treatment guidelines, protocols and standard operating procedures | - Has the radiotherapy management board reviewed current evidence and developed or adopted relevant clinical practice guidelines? - Has the radiotherapy management board considered its role in regularly updating the guidelines? - Has the radiotherapy service delivery process been defined? - Has a quality assurance policy been developed? |  |  |
| - 1. Other essential health services | - Are health services, such as laboratory, pathology and imaging services readily available in the LMIC? - Have providers of different cancer care such as surgery been identified? - Are there any comparable radiotherapy centres in the LMIC that are exemplars? |  |  |
| - 1. Social support services | - Is there assistance from the government to help introduce social services for cancer patients and their families? - Is there evidence of collaboration between the government and civil society organisations with the capacity to help with the provision of social services such as transport, meals and home help? |  |  |
| - 1. Generate, compile, analyse and communicate health data | - Have enough funds been allocated to develop, implement and operate information management tools? - Have qualified information technologists been identified and prepared for positions of responsibility? - Do the information communication technology staff have the experience to develop and implement safety and security procedures for managing patients’ records and information? - Has the LMIC considered its role in protecting patients’ data? |  |  |

**CATALYST**

**Domain 4**

| **Requirements** | **Questions** | **What is known (evidence)?** | **Next steps (actions)** |
| --- | --- | --- | --- |
| - 1. Encourage cancer control reform | - Is there support and recognition from senior political and health leaders that establishing a radiotherapy service addresses an important gap in cancer control and improves equity in cancer treatment access? - Is there a plan to use the new radiotherapy service as a window of opportunity to promote and mobilise resources to develop a comprehensive cancer service and optimise all aspect of cancer control? - Is there a plan to use the new radiotherapy service to advocate and create awareness about the growing burden of cancer and the importance of integrated cancer control strategies for managing it? |  |  |
| - 1. Promote coordinated care | - Are mechanisms such as nurse coordinator in place to take full advantage of the benefits of a coordinated cancer care? - Is there a relationship between service providers to facilitate effective communication and cancer patient referral to improve transition across specialists? - Are there information technology resources and policies that support coordination and continuity of care? |  |  |
| - 1. Strengthen patient- and family-centred care | - Are cancer patients and their families engaged in meaningful interactions to develop an accurate treatment plan based on available medical information? - Are cancer patients given access to the relevant information they need to make informed decisions? - Is there evidence to develop or adapt educational materials to support self-management? - Is there evidence of collaboration between the radiotherapy centre and civil society organisations to promote awareness about the benefits of radiotherapy services? |  |  |
| - 1. Promote a multidisciplinary approach to care | - Does the multidisciplinary oncology team (MDT) have the appropriate composition, with the right mix of specialist skills and experience to take responsibility of the cancer patient care planning? - Do the MDT members have a keen interest to communicate, manage and learn from others to effectively address patients’ needs? - Is there a chairperson with strong leadership to promote mutual professional respect among members of the MDT? - Is clinical information adequately shared to help MDT members understand the health problem prior to the meeting? - Are MDT members committed to regular meetings? - There is a process for communicating treatment outcomes and recommendations to patients and their general practitioners? - Is there evidence of an auditing mechanism to review the MDT functionality such as communication among MDT members and patients, meeting attendance and outcomes? |  |  |
| - 1. Encourage better outcomes through research | - Does the LMIC understand the value of oncology research? - Is there evidence of collaboration between the radiotherapy centre in the LMIC and different centres (international and/or regional) to improve oncology research? - If not, is there a plan to establish collaboration between the radiotherapy centre in the LMIC and different centres (international and/or regional) to improve oncology research? - Has the focus of the research collaboration clearly defined? - Have funds been allocated for research? |  |  |
